# Associations between four insulin resistance (IR) surrogates and the risk of small cell lung cancer (SCLC)

**DOI:** 10.1038/s41598-025-09548-0

**Published:** 2025-07-09

**Authors:** Yuedong Wang, Kun Zhang, Zhifei Xin, Wenjian Hu, Wenbo Wu, Yi Ma, Di Yao, Mutong Wang, Xiaopeng Zhang

**Affiliations:** 1 Hebei Medical University, Shijiazhuang, Hebei China; 2 Department of Thoracic Surgery, Hebei General Hospital, Shijiazhuang, Hebei China; 3 North China University of Science and Technology, Tangshan, Hebei China; 4 Hebei North University, Zhangjiakou, Hebei China; 5 Department of Cardiothoracic Surgery, Hebei General Hospital, Shijiazhuang, Hebei China

**Keywords:** Insulin resistance, Small cell lung cancer, Lung cancer, Oncology

## Abstract

Insulin resistance (IR) has been shown to be correlated with increased cancer risk. Nevertheless, few studies have explored the relationship between IR and small cell lung cancer (SCLC). The triglyceride glucose (TyG) index, TyG index with body mass index (TyG-BMI), triglyceride/high-density lipoprotein cholesterol ratio (TG/HDL-C), and metabolic score for IR (METS-IR) are recognized as reliable indicators for evaluating IR. In our investigation, 235 patients with pathologically confirmed SCLC were enrolled, along with 235 healthy individuals matched for age and sex as controls. Univariate binary logistic regression analyses revealed a significant association between elevated levels of all IR surrogates and the risk of SCLC. This finding persisted even after adjusting for other established high-risk factors. Concurrently, a progressive increase in the incidence of SCLC was observed across the tertiles of the TyG index, TyG-BMI, TG/HDL-C, and METS-IR. Furthermore, this article is the first to conclude that the four IR surrogates did not significantly differ across different stages of SCLC, implying that IR might exert a greater influence on the onset than on the progression of SCLC. Among these factors, TG/HDL-C has emerged as the most effective predictor of SCLC. Consequently, lifestyle modifications and pharmacological interventions should be actively pursued in individuals with IR to mitigate their risk of developing SCLC. Our findings also offer a promising avenue for the identification of novel therapeutic targets.

## Introduction

The 2022 Global Cancer Report (GLOBOCAN) highlights that lung cancer is the predominant form of cancer, with an estimated 2.48 million new cases (12.4%), and continues to be the prime culprit behind cancer-related fatalities, resulting in an estimated 1.82 million deaths (18.7%)^1^. Moreover, the International Agency for Research on Cancer (IARC) estimates that lung cancer is the most prevalent malignancy (22.0%) and the leading cause of cancer-related deaths (18.7%) in China^1^. Lung cancer can be categorized into two histological subtypes: non-small cell lung cancer (NSCLC) and SCLC. SCLC accounts for approximately 15% of all lung cancer cases. However, it is marked by a high propensity for recurrence and a poor survival rate, rendering it one of the most therapeutically challenging diseases in clinical practice^2^. Cigarette smoking has been identified as a major risk factor for SCLC, but the incidence of SCLC is also increasing in nonsmokers. Previously, research reported that 94% of SCLC patients in Western countries are smokers^3^. However, over 10% of Asian SCLC patients have never smoked, and known non-tobacco risk factors (such as secondhand smoke, radon exposure) cannot be universally intervened upon, nor can they explain all cases^4^. Additionally, the aggressiveness of SCLC is characterized by late diagnosis and limited treatment options, necessitating the proactive identification of actionable biological pathways for prevention. Therefore, there is an urgent need to investigate biologically modifiable mechanisms, such as IR, to supplement existing risk models.

IR is a condition in which cells and tissues are insensitive to the peptide hormone insulin^5^. Hyperinsulinaemia is characteristic of IR and promotes cell proliferation^6^. Numerous recent studies have demonstrated a robust association between IR and cancer risk and survival outcomes in patients with breast, thyroid, and endometrial cancers ^7–9^. However, studies on the relationship between NSCLC and IR remain controversial, as positive or invalid associations have been reported^10,11^. The hyperinsulinaemic-euglycaemic clamp test is regarded as the “gold standard” for assessing IR. However, it is infrequently employed in clinical settings because of the intricacy of the equipment, the complexity of the procedure, and its invasive nature^12^. Therefore, more practical alternative markers of IR, such as the TyG index, TyG-BMI, TG/HDL-C, and METS-IR, have been developed to replace direct measurement of IR^13^. Owing to its relatively low incidence, research on the correlation between SCLC and IR is still in its infancy. Hence, we aimed to investigate the link between IR and the risk of SCLC and to identify the most effective IR surrogate as a predictor of SCLC risk.

Previous research literature has primarily focused on non-lung cancer cancers or NSCLC, and this study fills the research gap on the association between SCLC and IR. At the same time, this study employed a case–control design, which is more suitable for etiological exploration of low-incidence cancers like SCLC, and reduced the interference of confounding factors by matching age and gender. In addition, this study first analyzed the differences in IR surrogates among the various stages (limited stage and extensive stage) of SCLC.

### Participants and methods

#### Participants

We retrospectively analysed data from 235 patients with newly diagnosed and pathologically confirmed SCLC at Hebei General Hospital between 2016 and 2024. For each SCLC patient, a sex- and age-matched healthy individual was enrolled as a control. Subjects with a history of cancer, diabetes, administration of lipid-lowering medications (e.g., atorvastatin and fenofibrate), or a history of hepatic or renal diseases impacting lipid metabolism (e.g., nephrotic syndrome) were excluded from the study. The research was conducted in accordance with the Declaration of Helsinki. All patients read and signed the written consent form. The study protocol was approved by the Hebei General Hospital Ethics Committee (NO.2025-LW-0007).

#### Physical examination and biochemical tests

The weights and heights of all the participants were measured via standardized stadiometers and scales while they were dressed in light clothing and without shoes. The smoking habits, medical history, including hypertension and cancer, and medication use data were obtained from the medical records collected upon admission. Venous blood samples were drawn from all participants in the morning following at least 10 h of fasting, and these samples were used to measure routine biochemical indicators. Fasting blood glucose (FBG) and lipid profiles, including total cholesterol (TC), triglyceride (TG), high-density lipoprotein cholesterol (HDL-C), low-density lipoprotein cholesterol (LDL-C), and uric acid levels, were quantified via commercial kits and analysed via an automated chemistry analyser (Model: TBA-FX8). Routine blood tests, such as white blood cell count (WBCC) and neutrophil count, were performed via an automated blood cell analyser (Model: SYXMEX XN-10). The following formula was used to calculate the four IR surrogates: TyG index = ln [triglyceride (mg/dL) × FPG (mg/dL) / 2]^14^. Additionally, TyG-BMI = TyG × BMI^15^, TG/HDL-C = triglycerides divided by high-density lipoprotein cholesterol^16^, and METS-IR = ln [(2 × FPG) + triglycerides] × BMI/ln (HDL-C)^17^.

#### Statistical analysis

Continuous variables with a normal distribution are presented as the means ± standard deviations and were compared via t tests. Variables exhibiting a skewed distribution were delineated by their median (p25-p75) and compared via nonparametric tests. Categorical variables are presented in terms of counts (percentages) and were compared via the chi-square test. Binary logistic regression was employed to explore the associations between the risk of SCLC and four IR surrogates while adjusting for potential confounders such as hypertension and smoking status. Receiver operating characteristic (ROC) analyses were performed to calculate the area under the ROC curve (AUROC) of the four IR surrogates for the incident of SCLC. The data analysis was executed with SPSS version 27.0.1 statistical software, with statistical significance defined as *P* < 0.05. R 4.4.2 was used for restricted cubic spline analysis and graphic plotting.

### Result

#### Baseline characteristics of the study population

The demographic and clinical characteristics of all the participants are listed in Table 1. The study population comprised 235 pathologically validated cases of SCLC and 235 age- and sex-matched healthy controls. There were significant differences in WBCC, neutrophil count, FBG, TG, HDL-C, and the four IR surrogates between the two groups. The incidence of smoking and hypertension was substantially greater in the SCLC cohort than in the control cohort. However, there were no significant differences in BMI, TC, LDL-C, or uric acid between the two groups.

**Table 1:** Demographic and clinical characteristics of all participants.

	Controls (n = 235)	SCLC (n = 235)	*P* value
Age (years)	65(59–71)	65(59–71)	1
Sex (Male/Female)	55/180	55/180	1
Smoking (%)	48(20.4%)	145(61.7%)	< 0.001
Hypertension (%)	46(19.6%)	102(43.4%)	< 0.001
BMI (kg/m2)	24.28 ± 3.15	24.86 ± 3.44	0.06
WBCC (× 10^9/L)	5.51(4.72–6.83)	6.63(5.49–8.09)	< 0.001
Neutrophil count (× 10^9/L)	3.72(2.91–4.80)	4.54(3.51–5.82)	< 0.001
FBG (mmol/L)	5.07 ± 0.67	5.36 ± 1.17	0.001
TC (mmol/L)	4.63 ± 0.78	4.61 ± 0.95	0.805
TG (mmol/L)	0.82 ± 0.21	1.18 ± 0.57	< 0.001
LDL-C(mmol/L)	2.88 ± 0.60	2.99 ± 0.72	0.084
HDL-C(mmol/L)	1.28(1.13–1.54)	1.14(0.96–1.36)	< 0.001
Uric acid (umol/l)	293.56 ± 78.32	277.40 ± 101.96	0.055
TyG index	8.06 ± 0.31	8.42 ± 0.48	< 0.001
TyG-BMI	195.98 ± 28.27	209.81 ± 34.23	< 0.001
TG/HDL-C	1.47 ± 0.51	2.49 ± 1.55	< 0.001
METS-IR	34.44 ± 5.54	37.47 ± 6.44	< 0.001

BMI, body mass index; WBCC, white blood cell count; FBG, fasting blood glucose; TC, total cholesterol; TG, triacylglyceride; LDL-C, low-density lipoprotein cholesterol; HDL-C, high-density lipoprotein cholesterol; TyG index, triglyceride‒glucose index; TyG-BMI, TyG index with body mass index; TG/HDL-C, triglyceride/high-density lipoprotein cholesterol ratio; METS-IR, metabolic score for insulin resistance.

#### Relationship between SCLC risk and four IR surrogates according to binary logistic regression analysis

Univariate binary logistic regression analyses revealed a significant association between elevated levels of the four IR surrogates and the risk of SCLC. In Model 2, following adjustment for hypertension and smoking, the four IR markers continued to show a significant association with SCLC risk. Furthermore, in Model 3, after accounting for smoking, hypertension, white blood cell count (WBCC), and neutrophil count, an elevated TyG index, TyG-BMI, TG/HDL-C, and METS-IR were indicative of a significantly increased risk of SCLC (Table 2).

**Table 2:** Logistic regression analysis of the four IR surrogates and SCLC risk.

	Model 1			Model 2			Model 3		
	OR	95%CI	*P*	OR	95%CI	*P*	OR	95%CI	*P*
TyG index	11.532	6.390–0.813	< 0.001	9.685	5.066–18.516	< 0.001	10.645	5.481–20.673	< 0.001
TyG-BMI	1.014	1.008–1.020	< 0.001	1.012	1.005–1.019	< 0.001	1.012	1.005–1.019	< 0.001
TG/HDL-C	4.358	3.089–6.148	< 0.001	3.625	2.513–5.231	< 0.001	3.618	2.500–5.236	< 0.001
METS-IR	1.089	1.054–1.124	< 0.001	1.065	1.028–1.104	< 0.001	1.063	1.025–1.102	0.001

Model 1: unadjusted.

Model 2: adjustment for smoking and hypertension.

Model 3: adjustment for smoking, hypertension, WBCC, and neutrophil count.

#### The incidence of SCLC compared across the tertiles of the four IR surrogates

**Fig. 1:**
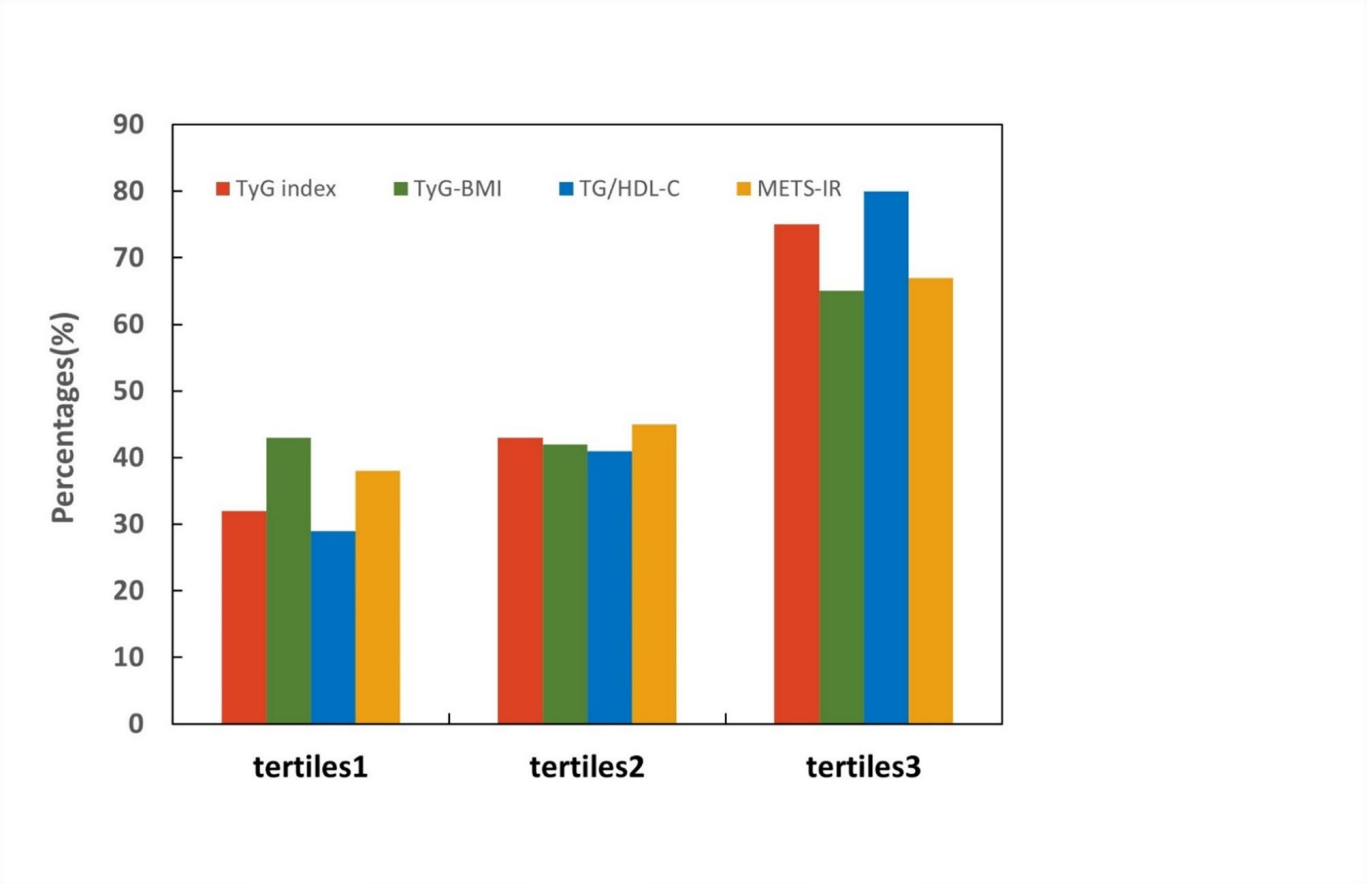
Incidence of SCLC compared across the tertiles of the four IR surrogates.

All of our included participants were divided into three groups on the basis of the tertiles of the four IR surrogates’ levels (tertiles1: levels ≤ 33.3rd percentile, tertiles2: levels > 33.3rd and ≤ 66.7th percentile, tertiles3: levels > 66.7th percentile), and their incidence of SCLC was calculated separately; the results are shown in Fig. 1. Along the tertiles of the TyG index, TyG-BMI, TG/HDL-C and METS-IR, a continued increase in the incidence of SCLC was observed (TyG index: 32%, 43%, 75%; TyG-BMI: 43%, 42%, 65%; TG/HDL-C: 29%, 41%, 80%; METS-IR: 38%, 45%, 67%. *P* < 0.001 all).

#### The four IR surrogates in different stages of SCLC

Patients with SCLC were divided into 2 groups according to stage: limited stage (n = 140) and extensive stage (n = 95). The TyG index, TyG-BMI, and METS-IR exhibited normal distribution patterns, whereas the TG/HDL-C followed a skewed distribution between the two groups. Patients in the limited stage presented higher TyG index, TyG-BMI, TG/HDL-C, and METS-IR values than did those in the extensive stage; however, no statistically significant differences were detected between the two cohorts, implying that IR might exert a greater influence on the onset than on the progression of SCLC (Table 3).

**Table 3:** The four IR surrogates in different stages of SCLC.

	Limited stage (n = 140)	Extensive stage (n = 95)	*P* value
TyG index	8.45 ± 0.48	8.38 ± 0.47	0.290
TyG-BMI	212.65 ± 34.54	205.63 ± 33.51	0.123
TG/HDL-C	2.14(1.60–3.11)	2.13(1.40–2.61)	0.336
METS-IR	37.91 ± 6.63	36.82 ± 6.12	0.203

#### The value of the four IR surrogates for predicting the incident of SCLC

**Fig. 2:**
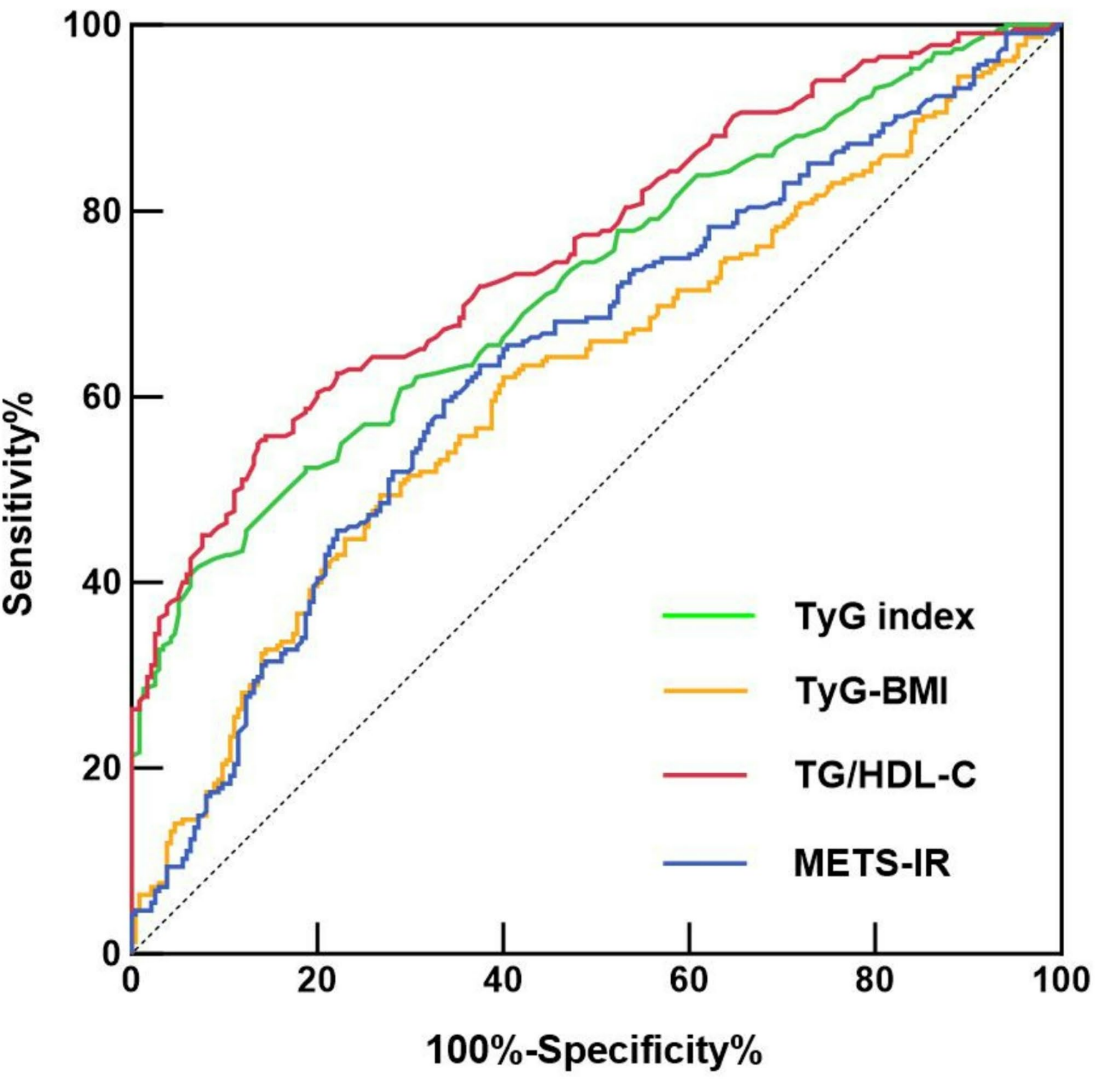
Receiver operative characteristic (ROC) curves of the four IR surrogates for predicting the incident of SCLC.

ROC analysis revealed that the TyG index predicted the occurrence of SCLC with an AUROC of 0.7228 (95% CI 0.6774–0.7682, *P* < 0.0001), and the optimal cut-off value for the TyG index was 8.255 (sensitivity: 60.85%, specificity: 71.06%). Concurrently, the AUROCs for TyG-BMI, TG/HDL-C, and METS-IR in predicting SCLC were 0.6188 (95% CI 0.5681–0.6695, *P* < 0.0001), 0.7600 (95% CI 0.7173–0.8026, *P* < 0.0001), and 0.6402 (95% CI 0.5902–0.6902, *P* < 0.0001), respectively, with optimal cut-off points of 197.6 (sensitivity: 62.13%, specificity: 60.00%), 1.855 (sensitivity: 62.55%, specificity: 77.87%), and 35.56 (sensitivity: 60.43%, specificity: 65.11%) (Fig. 2). Therefore, we concluded that TG/HDL-C was the most accurate predictor of SCLC among these IR surrogates.

#### Dose–response relationship between the four IR surrogates and SCLC

**Fig. 3:**
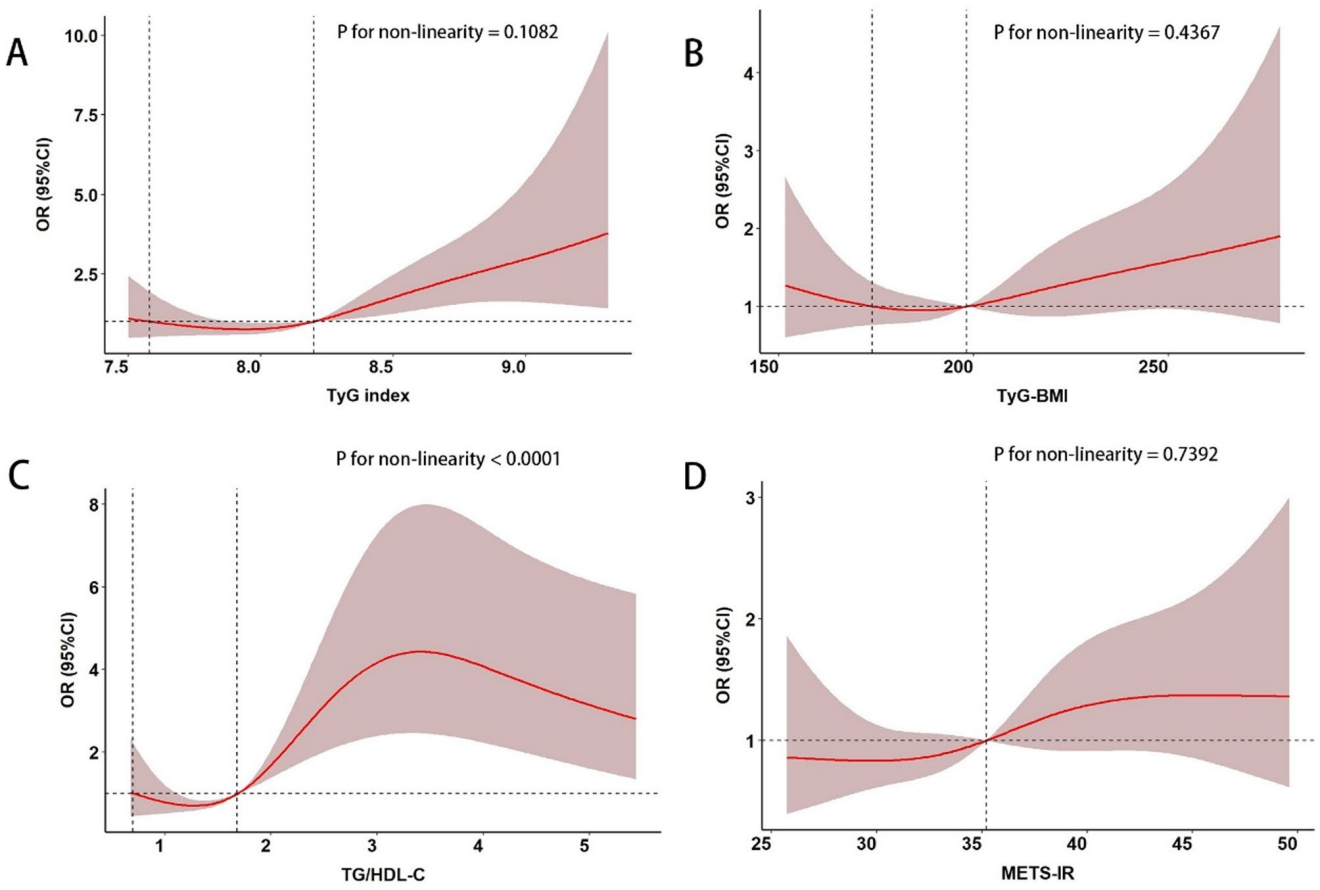
Dose–response relationship between the four IR surrogates and SCLC.

After using restricted cubic splines and adjusting for relevant confounders, there was a linear dose–response relationship between the TyG index (P non-linearity = 0.1082), TyG-BMI (P non-linearity = 0.4367), and METS-IR (P non-linearity = 0.7392) with the risk of SCLC, while there was a non-linear dose–response relationship between TG/HDL-C (P overall < 0.05, P non-linearity < 0.0001) and the risk of SCLC (Fig. 3).

## Discussion

SCLC is a malignant neuroendocrine malignancy that occurs mainly in the central airways of heavy smokers^18^. SCLC is characterized by the gradual onset of symptoms before extensive metastasis, rapid growth, high initial response rates to chemotherapy, strong potential for early dissemination, and drug resistance^19^. Compared with NSCLC, screening for SCLC via computed tomography does not improve patient outcomes^20^. Owing to the aforementioned characteristics of SCLC, the vast majority of cases are confirmed when the disease is advanced and unresectable. The loss of surgical opportunities leads to insufficient biopsy samples, which limits clinical research into the biology of SCLC and the development of therapeutic approaches^18^. Owing to the lack of existing treatment options, the prognosis for SCLC patients remains poor. The development of SCLC is usually associated with overexposure to tobacco, with one study showing that 94% of men and 93.9% of women with SCLC had ever smoked^3^. Interestingly, the proportion of SCLC patients who have never smoked appears to be slightly greater among Asians, with more than 10% of them never having smoked^4,21^. Notably, secondhand smoke also contributes to the development of SCLC, as the association between secondhand smoke exposure and the development of SCLC is two to three times greater than that for other histological types^22^. Indoor radon exposure and air pollution may be the second most important risk factor for SCLC globally and the primary risk factor for never-smokers^23,24^. Among respiratory comorbidities, chronic obstructive pulmonary disease (COPD) is an independent risk factor for the development of SCLC^25^. Beyond tobacco-related factors, the current gaps in understanding the etiology of SCLC are particularly concerning, especially for those who have never smoked and former smokers who still face persistent risk. Although environmental exposures contribute to the development of SCLC, these factors are difficult to modify at the individual level. In contrast, IR represents an actionable biological target. Its role in promoting carcinogenesis through various mechanisms has been gradually clarified, and IR can be screened using cost-effective surrogates and modulated through lifestyle interventions or medications. Our findings demonstrate that IR surrogates independently predicted the risk of SCLC, even after adjusting for smoking and inflammatory factors, reinforcing the potential of IR as a novel modifiable risk factor.

Substantial evidence indicates that IR is associated with the occurrence of various cancers, including lung, prostate, colorectal, and breast cancers, suggesting that IR may serve as an effective tool for identifying individuals at risk for cancer. Our study provides an in-depth look at the associations between four IR surrogates and the risk of SCLC in the Chinese population. The results revealed significant differences in WBCC, neutrophil count, FBG, TG, HDL-C, and the four IR surrogates between the two groups. The prevalence of smoking and hypertension was significantly greater in the SCLC group than in the control group. When we corrected for the traditionally known high risks of smoking, hypertension, WBCC, and neutrophil count, higher TyG index, TyG-BMI, TG/HDL-C, and METS-IR values were associated with a greater risk of developing SCLC. Along the tertiles of the four IR surrogates, a continued increase in the incidence of SCLC was observed. The four IR surrogates did not significantly differ across the different stages of SCLC (limited stage and extensive stage). We concluded that TG/HDL-C was the best predictor of SCLC among these IR surrogates by ROC analysis. The AUROC of TG/HDL-C in predicting SCLC occurrence was 0.7600 (95% CI 0.7173–0.8026, *P* < 0.0001), and the optimal cut-off point was 1.855 (sensitivity: 62.55%, specificity: 77.87%). These results suggest that IR is a high-risk factor for the development of SCLC. TG/HDL-C is a simple and effective predictor of SCLC risk. The increased risk of SCLC in people with IR should be considered, and lifestyle interventions or pharmacological treatments should be given in advance. This would effectively reduce the burden of cancer expenditures on the state and government.

IR can increase cancer risk through several pathways. IR is a condition characterized by the reduced biological activity of insulin, leading to an increase in insulin secretion to compensate for its diminished function^26^. However, with increasing insulin secretion, the secretion of insulin-like growth factors (IGFs) also increases^27^. IGFs, which include IGF-1 and IGF-2, are a group of peptide hormones that can promote various cellular processes, such as cell proliferation, migration, invasion, and epithelial‒mesenchymal transition. IR not only has a significant effect on the biological characteristics of tumor cells but also has a profound effect on the tumor microenvironment, thereby promoting the development of lung cancer^28^. The cellular insulin response is compromised, affecting glucose metabolism and cell proliferation. Owing to the inability of cells to utilize glucose effectively as an energy source, their demand for alternative nutrients, such as fatty acids and amino acids, increases to compensate for the energy deficit. This metabolic shift creates a more favourable environment for tumor cell survival, growth, and metastasis. In addition, an enhanced inflammatory response under IR is associated with the progression of lung cancer^26^. This study revealed that IR can stimulate the production and release of inflammatory factors by activating inflammatory-related signalling pathways. These inflammatory factors contribute to the promotion of tumor cell growth, invasion, and metastasis^29,30^. Moreover, current research suggests that IR can lead to reactive oxygen species (ROS) production through several potential pathways. The overproduction of ROS overwhelms cellular antioxidant defense mechanisms, ultimately leading to cellular damage. Studies have shown that ROS can lead to deoxyribonucleic acid (DNA) damage, gene mutation, and apoptosis, all of which are important factors in the development of tumours^31^. Under conditions of IR, T-cell function is suppressed, leading to a decreased ability to recognize and eliminate tumor cells^26^. Further studies of these mechanisms will help us discover more therapeutic targets and shed new light on SCLC patients.

There are several limitations to this study. First, owing to the cross-sectional design, it is difficult to determine whether the TyG index, TyG-BMI, TG/HDL-C, and METS-IR have causal effects on SCLC. Second, the results revealed that the correlation between the four IR surrogates and SCLC risk remained significant after adjusting for indicators of inflammation, including WBCC and neutrophil count; however, since other indicators of inflammation, such as C-reactive protein and tumor necrosis factor-α (TNF-α), were not detected and analysed in this study, the potential role of IR in the risk of inflammation-induced SCLC needs to be further investigated. Some other risk factors (e.g., secondhand smoke, indoor radon exposure, air pollution, COPD) were not corrected for when the regression analyses were performed. Finally, multicentre and large-scale prospective studies are needed to confirm our results.

## Conclusions

IR can increase the risk of SCLC. TG/HDL-C was the most accurate predictor of SCLC among the four IR surrogates. Lifestyle interventions or pharmacological treatments should be actively pursued in people with IR to reduce their risk of SCLC. Our findings also provide a potential avenue for identifying new therapeutic targets.

## Data Availability

The datasets used and/or analyzed during the current study are available from the corresponding author upon reasonable request.
